# Evaluation of Virtual Grid processed clinical pelvic radiographs

**DOI:** 10.1002/acm2.14353

**Published:** 2024-05-01

**Authors:** Tim Gossye, Dimitri Buytaert, Peter V. Smeets, Lieve Morbée, Elke Vereecke, Pieter‐Jan Kellens, Eric Achten, Klaus Bacher

**Affiliations:** ^1^ Department of Human Structure and Repair Ghent University Ghent Belgium; ^2^ Department of Radiology Ghent University Hospital Ghent Belgium

**Keywords:** emergency, intensive care unit, pelvic region, radiography, scatter

## Abstract

**Background:**

A physical scatter grid is not often used in pelvic bedside examinations. However, multiple studies regarding scatter correction software (SC SW) are available for mobile chest radiography but the results are unclear for pelvic radiography.

**Purpose:**

We evaluated SC SW of Fujifilm (Virtual Grid) on gridless pelvic radiographs obtained from a human Thiel‐embalmed body to investigate the potential of Virtual Grid in pelvic bedside examinations.

**Methods:**

Gridless, Virtual Grid, and physical grid pelvic radiographs of a female Thiel‐embalmed body were collected with a broad range of tube loads. Different software (SW) grid ratios—6:1, 10:1, 13:1, 17:1, and 20:1—were applied on the gridless radiographs to investigate the image quality (IQ) improvement of 13 IQ criteria in a visual grading analysis (VGA) setup.

**Results:**

Gridless radiograph scores are significantly lower (*p* < 0.001) than Virtual Grid and physical grid scores obtained with the same tube load. Virtual Grid radiographs score better than gridless radiographs obtained with a higher tube load which makes a dose reduction possible. The averaged ratings of the IQ criteria processed with different SW ratios increase with increasing SW grid ratios. However, no statistically significant differences were found between the SW grid ratios. The scores of the physical grid radiographs are higher than those of the Virtual Grid radiographs when they are obtained with the same tube load.

**Conclusion:**

We conclude that Virtual Grid with an SW ratio of 6:1 improves the IQ of gridless pelvic radiographs in such a manner that a dose reduction is possible. However, physical grid radiograph ratings are higher compared to those of Virtual Grid radiographs.

## 1 INTRODUCTION

A physical scatter rejection grid is often not used in pelvic bedside examinations due to the occurrence of misalignment artifacts when the grid is not properly aligned with the x‐ray focal spot. The use of a scatter rejection grid increases the patient dose, typically 2−6 fold, when the detector air kerma (DAK) remains constant.^1^, ^2^ As an alternative to physical scatter rejection grids manufacturers released scatter correction software (SC SW) to improve the image quality (IQ) of radiographs.^3^, ^4^, ^5^, ^6^, ^7^, ^8^, ^9^ Fujifilm (Tokyo, Japan) is one of the manufacturers with SC SW —Virtual Grid— to improve the radiographs that are obtained without a physical grid (gridless). Recently published papers about Virtual Grid evaluate the performance in the chest region. In these studies, good results are observed in favor of Virtual Grid, but none are directly linked to the pelvis.^9^, ^10^, ^11^, ^12^

The aim of the study was to compare the Virtual Grid SW pelvic radiographs obtained with multiple‐dose settings with the gridless and physical grid pelvic radiographs.

## 2 MATERIALS AND METHODS

### 2.1 Image acquisition

A female Thiel‐embalmed body was selected for the study with body length of 165 cm and weight of 68 kg after embalming (BMI: 25 kg/m^2^).

The radiographs were obtained with a fixed radiography system (Siemens Ysio, Optitop 150/40/80 HC‐100; large focal spot size 1.0 mm and small focal spot 0.6 mm) with a removable stationary focused grid (ratio 15:1, line pairs (lp)/cm: 80, interstitial material: Al, focus‐detector range: 115−180 cm, focus length: 115 cm). The source‐to‐image distance was set to 115 cm as programmed by Siemens, the source‐to‐patient table distance was 110 cm, and the source‐to‐patient entrance surface was 93 cm.

To obtain the radiographs, a CsI flat panel detector (FDR D‐EVO II C35, Fujifilm, Tokyo, Japan) was inserted into the detector tray. The active area of the detector was 425.4 mm × 350.4 mm with a pixel pitch of 0.150 mm, resulting in 2836 × 2336 pixels.

Every radiograph was obtained without and with 0.1 mm Cu in the collimator and a selected tube voltage of 81 kV with different tube loads. The tube loads chosen by the radiographic system on automatic exposure cells (AEC) were 2 mAs without a grid, and 10 mAs with a grid, both with 0.1 mm Cu. Table 1 presents the tube loads, the entrance surface doses (ESDs), and the DAKs to which the Thiel‐embalmed body was exposed. All presented ESDs were measured with an ionization chamber (Radcal Corporation Model 10 × 6‐6, Monrovia, CA USA, 2017), placed on the surface of the Thiel‐embalmed body in the central axis of the x‐ray beam. The DAKs were measured with a solid‐state detector (Radcal Corporation Model AGMS‐DM+ Multisensor; Diag/Mammo, Monrovia, CA, 2017).

**TABLE 1:** Dose information of the obtained radiographs.

	Gridless and Virtual Grid radiographs					
Tube load (mAs)	2	3.6	5	7.1	10	12.5
Entrance surface dose (mGy) (without/with Cu)	0.14/0.07	0.26/0.13	0.36/0.18	0.51/0.26	0.72/0.37	0.90/0.46
Detector air kerma (µGy) (without/with Cu)	2.9/2.3	5.4/4.3	7.6/6.0	10.8/8.5	15.2/12.0	19.1/15.1
SW grid ratios	0 (SC SW off), 6:1, 10:1, 13:1, 17:1, and 20:1					
Cu filtration	0.0 and 0.1 mm Cu					

	Physical grid radiographs					
Tube load (mAs)	2	3.6	5	7.1	10	12.5
Detector air kerma (µGy) (without/with Cu)	0.6/0.5	1/0.9	1.4/1.2	2/1.6	2.8/2.3	3.6/2.9
Cu filtration	0.0 and 0.1 mm Cu					

Afterwards, each radiograph that was obtained without a physical grid was processed with five different Virtual Grid SW grid ratios (6:1, 10:1, 13:1, 17:1, and 20:1) (version 13.0). The SW grid density and the SW grid interspace material were set to 40 lines/cm and fiber, respectively, as recommended by an application specialist of Fujifilm. In total, 84 radiographs were obtained. Twelve radiographs without any SC SW (gridless), 60 radiographs with SC SW, and 12 radiographs with a physical grid.

### 2.2 Image analysis and rating of the radiographs by human observers

Pelvis IQ criteria were identified by three experienced radiologists (36, 9, and 8 years of experience), who participated later in the relative visual grading analysis (VGA) study (Table 2). All radiographs were displayed on a 30‐inch 6 MP Barco screen (model: Coronis Fusion; Barco NV, Kortrijk, Belgium), which was configured in 2 × 3 MP. The reference image that was presented to the radiologists was obtained with 2 mAs, 0.1 mm Cu, and processed with a SW grid ratio of 10:1 (recommended SW grid ratio by an application specialist of Fujifilm). Each IQ criterion was rated on a 5‐point scale (−2 = much worse, −1 = worse, 0 = equal, +1 = better, +2 = much better) in SW for IQ evaluation (ViewDex 2.48).^13^ The radiologists received training before the study to get familiar with the software and the scoring method. The observers were not allowed to zoom in and adjust the image brightness and contrast during the study. After all the radiographs were evaluated, a relative VGA score was calculated for each radiograph and radiologist (1).

(1) $$VGA\;score\frac{\sum_{c\;=\;1}^{C}R_{rel,c}}{C\;}$$

where *C* represents the number of IQ criteria included in the formula (i.e., 13). R_rel,c_ is the relative rating of a certain criterion (1−13) (anatomical structures) from a certain radiograph.

**TABLE 2:** Overview of the different IQ criteria.

Criterion (C) number	Criterion (C)
1	Visually sharp reproduction of the
	trabecular pattern
2	delineation of the lateral wall of the ascending colon
3	left transverse process of L4
4	right transverse process of L4
5	left inferior articular process of L3
6	right inferior articular process of L3
7	left intermuscular fat planes between the gluteal muscles at the insertion
8	right intermuscular fat planes between the gluteal muscles at the insertion
9	sigmoidal bowel gas
10	coccygeal bone
11	anterior sacral foramina at s1
12	superior margin of the left sacroiliac joint
13	phleboliths of the small pelvis
14	Subjective perceived image noise
15	Is the image approved for diagnostic use

### 2.3 Statistical analysis

Analyses were done with Statistical Package for Social Sciences (SPSS) (IBM Corp Released 217, IBM SPSS Statistics for Windows, Version 25.0; IBM Corp, Armonk, NY). The reliability among the radiologists was determined with the intraclass correlation coefficient (ICC). Values between 0.75 and 0.90 are considered as good reliability.^14^ The effect of the variables on the human observer scores was investigated by using a linear regression. model in a similar way as earlier described.^11^ Model building with manual forward stepwise selection was applied to select the final model based on Akaike Information Criteria (AIC) starting from an univariate model. The following variables (effects) were selected: type of grid, the tube load, the ratings of the IQ criteria (C1–C13), and Cu filtration. In a similar way, we added two‐way interaction terms based on the smallest AIC. Interaction terms are useful to investigate the influence of one variable on the other variable for its effect on the outcome.

Similarly to the previous model building method, the effects of the different Virtual Grid SW grid ratios on the ratings of the IQ criteria (C1–C13) were more thoroughly investigated considering only Virtual Grid radiographs. Additionally, the noise criterion (C14) was investigated as well.

To correct for multiple comparisons, we applied the Bonferroni‐Holm correction to the *p* value (adjusted [adj.] *p* value). *p* values < 0.05 are considered statistically significant.

## 3 RESULTS

### 3.1 Image analysis and rating of the radiographs by human observers

**FIGURE 1:**
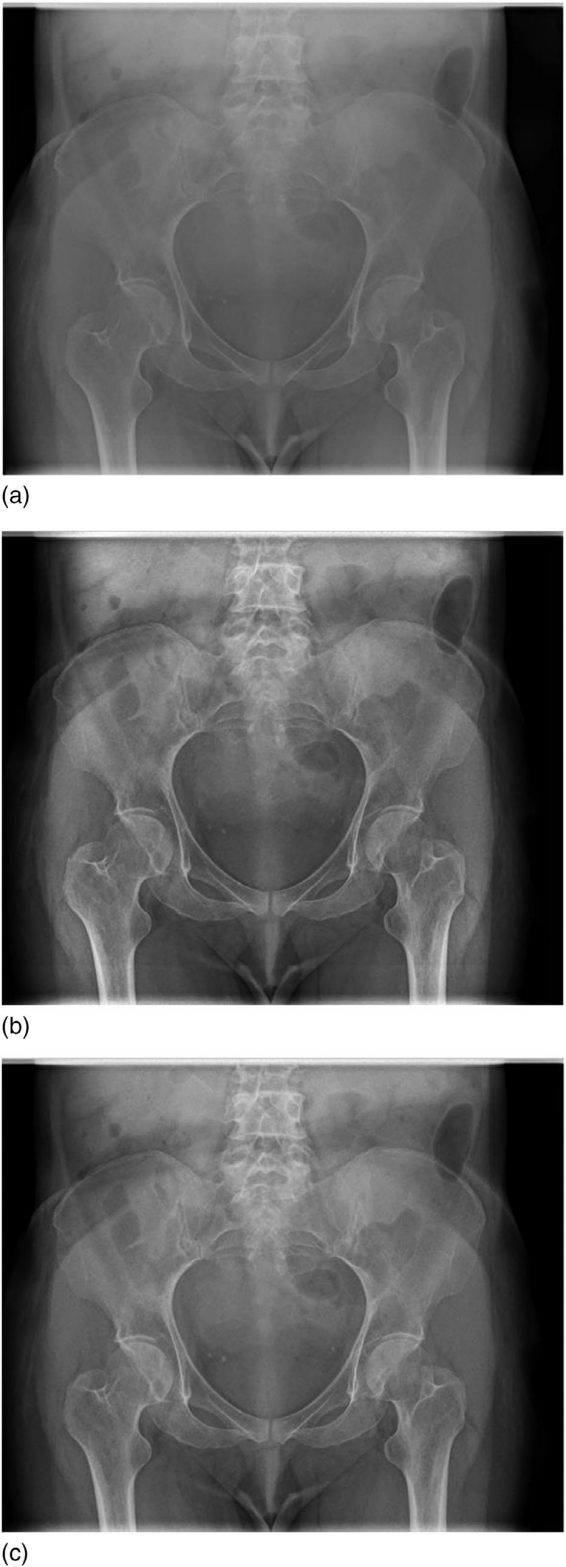
Example of the pelvic radiographs of the Thiel‐embalmed body. The presented radiographs were obtained with 81 kV, 2 mAs, and 0.1 mm Cu filtration. Figures (a–c) show the gridless, Virtual Grid SW ratio 10:1, and the physical grid radiographs, respectively.

The ICC was calculated with a two‐way mixed‐effects model, which resulted in an average ICC of 0.88. Therefore, averaged ratings of the IQ criteria across the three radiologists were used in further analysis. All the radiographs were approved for diagnostic use (C15). Examples of gridless, Virtual Grid SW ratio 10:1, and physical grid radiographs are presented in Figure 1.

In the final regression model the variables—type of grid (gridless, Virtual Grid, and physical grid), the tube load, the IQ criteria (C1–C13), and Cu filtration—were included as main effects after model building. The only interaction term that contributed to the model fit was the type of grid x criteria. Further analysis with multiple pairwise comparisons of the interaction term showed that the average ratings of all the gridless IQ criteria are significantly lower (*p *< 0.001) than the average ratings of the IQ criteria of Virtual Grid. The average ratings of all the IQ criteria, except criterion 12, of the Virtual Grid images processed with SW grid ratio 6:1 or 10:1 had no significant differences with the physical grid. No statistically significant differences were found for the average ratings of all IQ criteria between SW ratio 20:1 and physical grid. However, 10 (all, except C1, C8, C9, and C14) IQ criteria for Virtual Grid processed radiographs with SW ratio of 20:1 scored lower than the physical grid IQ criteria. The average ratings for each of the IQ criteria for gridless, Virtual Grid with SW ratios of 6:1−20:1, and physical grid images are presented in Figure 2. As there was no interaction between the tube loads and IQ criteria or Cu filtration, averages are presented in the latter figure.

FIGURE 2(a–c) Averaged ratings of the three radiologists per criterion (Table 2). Each criterion was scored for gridless, SW grid ratios 6:1, 10:1, 13:1, 17:1, 20:1, and physical grid images. The error bars represent the standard deviation of the ratings of the radiologists. The lowest averaged ratings are observed when the Virtual Grid ratio is off (SW ratio 0), except for the noise criterion (C14). This indicates that the perceived image noise was lower in the gridless images. C1:Visually sharp reproduction of the trabecular pattern, C2: Visually sharp reproduction of the delineation of the lateral wall of the ascending colon, C3: Visually sharp reproduction of the left transverse process of L4, C4: Visually sharp reproduction of the right transverse process of L4, C5: Visually sharp reproduction of the left inferior articular process of L3, C6: Visually sharp reproduction of the right inferior articular process of L3, C7: Visually sharp reproduction of the left intermuscular fat planes between the gluteal muscles at the insertion, C8: Visually sharp reproduction of the right intermuscular fat planes between the gluteal muscles at the insertion, C9: Visually sharp reproduction of the sigmoidal bowel gas, C10: Visually sharp reproduction of the coccygeal bone, C11: Visually sharp reproduction of the anterior sacral foramina at s1, C12: Visually sharp reproduction of the superior margin of the left sacroiliac joint, C13: Visually sharp reproduction of the phleboliths of the small pelvis, C14: Subjective perceived image noise.

**Figure:**
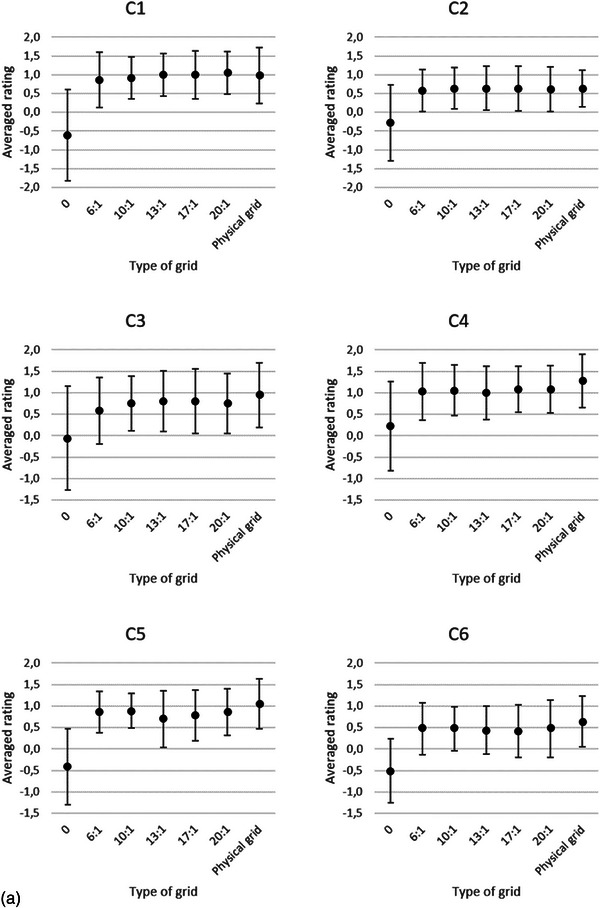

**Figure:**
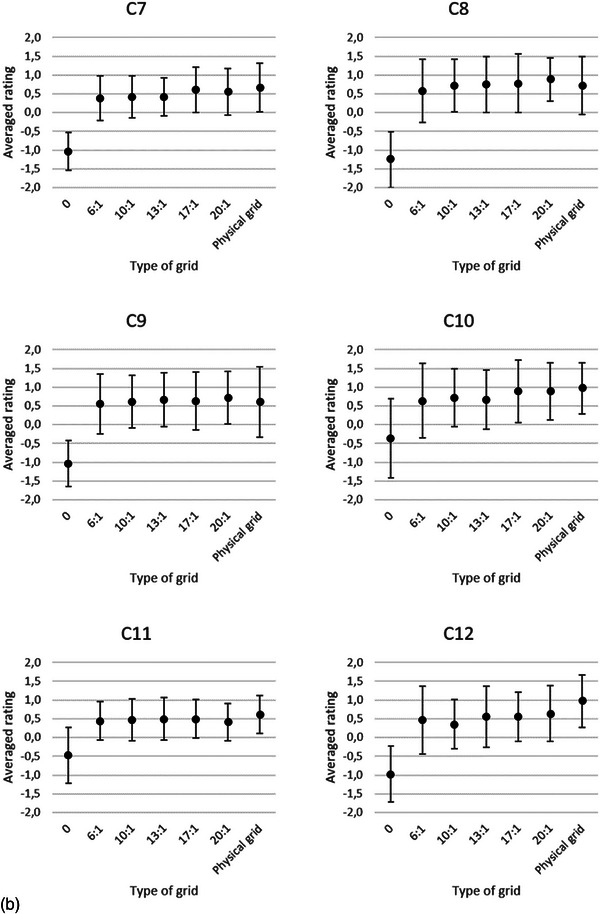

**Figure:**
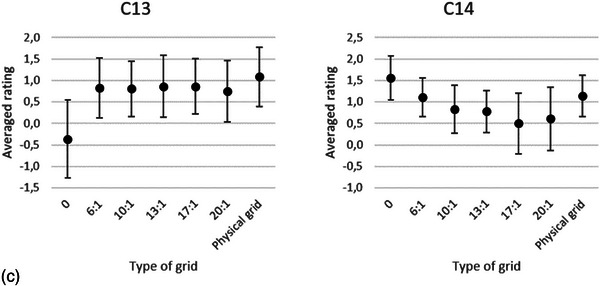

Considering only the Virtual Grid radiographs the average ratings of the IQ criteria ratios (6:1−20:1) were affected by the variables tube load, ratings of the IQ criteria, and Cu filtration. No second‐order interaction term SW grid ratio x IQ criteria (Figure 2) or significant differences (adj. *p *> 0.05) between the SW ratios (6:1−20:1) were found. The averaged VGA scores for SW ratios 0, 6:1, 10:1, 13:1, 17:1, and 20:1 are −0.55, 0.64, 0.68, 0.69, 0.74, and 0.75, respectively.

**FIGURE 3:**
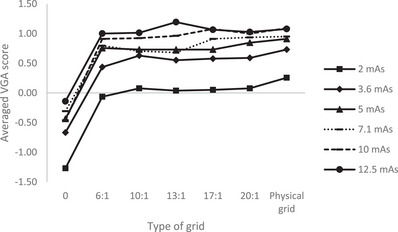
Averaged scores of the criteria C1–C13 in function of the type of grid for every used tube load. A higher tube load results in a better‐averaged rating. The images obtained with the physical grid generally result in a better rating, even with the lowest tube load of 2 mAs.

Figure 3 presents a more detailed observation of the averaged VGA scores of the images obtained with the different tube loads and types of grid. For every tube load, a significant difference was found in the averaged VGA scores between gridless and SW ratio ≥ 6:1, but no significant difference was found in the averaged VGA scores between SW ratio ≥ 6:1 and physical grid (adj. *p *> 0.05).

The model that includes only the noise criterion (C14) shows that the variables—type of grid, tube load, and the interaction term type of grid x tube load—were included as main effects after model building. Investigation of the pairwise comparisons of the latter showed that there were no significant differences noticed in noise between gridless and SW ratio 6:1 radiographs for every tube load. Significant differences were observed between gridless and SW ratio ≥ 10:1 for 2 and 3.6 mAs (adj. *p *< 0.05), but not for the higher tube loads.

There were no significant differences between SW ratio 6:1 and physical grid radiographs for all the tube loads, but there was a significant difference between the SW ratio 20:1 radiographs and physical grid radiographs obtained with 2 mAs (adj. *p *< 0.01). For the other tube loads, no significant differences were observed between the SW ratio of 20:1 and the physical grid

## 4 DISCUSSION

The averaged ratings of all the IQ criteria (Figure 2) and the linear regression model show that Virtual Grid radiographs processed with SW ratio ≥ 6:1 improve the IQ of gridless radiographs (adj. *p* < 0.001). A small increase in VGA scores is noticed with higher SW ratios, but in this study we did not find a significant result. This result is similar to the result of Gossye et al., where the IQF_inv_ of the CDRAD phantom increases with increasing SW ratios, as well as in the study where the VGA scores of chest radiographs increase with the SW ratios.^11^, ^12^ The latter study shows that a SW grid ratio of 6:1 improved the gridless averaged ratings of 12 chest IQ criteria (adj. *p* < 0.001). An important finding of the study of Gossye et al. on patient chest radiographs was the decrease in the visibility of the spine.^12^ This was attributed to the increase in noise in the latter structure when higher SW ratios were selected (> 13:1). In our study, we did not notice a pronounced decrease in any of the averaged ratings of the IQ criteria, which encompasses the spine. However, we noticed an increase in the amount of perceived noise in the radiographs when the SW ratios were increased.

In the current study, the scores of all IQ criteria (except C12) show no significant differences between the Virtual Grid SW ratios 6:1/10:1 and physical grid radiographs. Nevertheless, in Figure 3, the IQ scores of the physical scatter rejection grid are higher, even with the lowest tube load, that is, 2 mAs. This result is somehow unexpected as the tube load was 10 mAs determined on the AEC with 0.1 mm Cu filtration and with a physical grid inserted, resulting in a DAK of 2.3 µGy. The tube load of 2 mAs with Cu filtration and with a physical grid resulted in a DAK of only 0.5 µGy. The rating of the noise values of the latter was investigated. However, with an average rating of 0.83, the radiologists judged that the amount of subjective perceived noise was better than in the reference image that was processed with a SW ratio of 10:1. Furthermore, all radiographs were judged as clinically diagnostically acceptable.

The results from our study shows that a dose reduction with Virtual Grid is possible while maintaining IQ. From Figure 3, it is clear that the Virtual Grid radiograph obtained with 2 mAs and processed with a SW ratio of 6:1 results in a better‐averaged rating than the gridless radiographs that were obtained with higher mAs values.

The study contains some limitations. First, the study was performed with only one Thiel‐embalmed body with an average BMI of 25 kg/m^2^ after embalming. More embalmed bodies should be included with variation in BMI. In this way, also the statistical power will be increased. Second, IQ evaluation was performed in the current study by evaluating normal anatomy. The current results do not necessarily reflect task based scoring methods, for example, receiver operating characteristic.

Third, in this study the physical grid was perfectly aligned with the tube. In bedside examination conditions the alignment of the physical grid with the tube is difficult. Therefore, the ratings of the physical grid radiographs are most likely an overestimation in the current study. Fourth, the used physical grid had a grid ratio of 15:1, where the SW grid ratios was above and below the grid ratio of the physical grid.

We conclude that Virtual Grid with a SW ratio of 6:1 improves the IQ of gridless pelvic radiographs in a manner that a patient dose reduction is possible. No statistically significant difference was found between a SW ratio of 6:1 and the higher SW ratios, but the ratings of the radiologists increased with increasing SW ratio. The perceived amount of noise is less than when higher SW grid ratios are selected. Despite the use of a physical grid with relatively low tube loads, the ratings of the physical grid are higher than those of the Virtual Grid radiographs. However, the differences decrease with higher tube loads.

## AUTHOR CONTRIBUTIONS

All authors declare that they were involved in the conceptualization of the design, analyzing, interpreting the results, drafting and/or revising the work. All author's declare that they approved the version that is considered to be published. All author's Agree to be accountable for all aspects of the work in ensuring that questions related to the accuracy or integrity of any part of the work are appropriately investigated and resolved.

## CONFLICT OF INTEREST STATEMENT

The authors declare that they have no known competing financial interests or personal relationships that could have appeared to influence the work reported in this paper.
